# Cysteine Metabolism and Oxidative Processes in the Rat Liver and Kidney after Acute and Repeated Cocaine Treatment

**DOI:** 10.1371/journal.pone.0147238

**Published:** 2016-01-25

**Authors:** Danuta Kowalczyk-Pachel, Małgorzata Iciek, Karolina Wydra, Ewa Nowak, Magdalena Górny, Małgorzata Filip, Lidia Włodek, Elżbieta Lorenc-Koci

**Affiliations:** 1 The Chair of Medical Biochemistry, Jagiellonian University Medical College, Cracow, Poland; 2 Laboratory of Drug Addiction Pharmacology, Institute of Pharmacology, Polish Academy of Sciences, Cracow, Poland; 3 Department of Neuro-Psychopharmacology, Institute of Pharmacology, Polish Academy of Sciences, Cracow, Poland; University of Pecs Medical School, HUNGARY

## Abstract

The role of cocaine in modulating the metabolism of sulfur-containing compounds in the peripheral tissues is poorly understood. In the present study we addressed the question about the effects of acute and repeated (5 days) cocaine (10 mg/kg i.p.) administration on the total cysteine (Cys) metabolism and on the oxidative processes in the rat liver and kidney. The whole pool of sulfane sulfur, its bound fraction and hydrogen sulfide (H_2_S) were considered as markers of anaerobic Cys metabolism while the sulfate as a measure of its aerobic metabolism. The total-, non-protein- and protein- SH group levels were assayed as indicators of the redox status of thiols. Additionally, the activities of enzymes involved in H_2_S formation (cystathionine γ-lyase, CSE; 3-mercaptopyruvate sulfurtransferase, 3-MST) and GSH metabolism (γ-glutamyl transpeptidase, γ-GT; glutathione S-transferase, GST) were determined. Finally, we assayed the concentrations of reactive oxygen species (ROS) and malondialdehyde (MDA) as markers of oxidative stress and lipid peroxidation, respectively. In the liver, acute cocaine treatment, did not change concentrations of the whole pool of sulfane sulfur, its bound fraction, H_2_S or sulfate but markedly decreased levels of non-protein SH groups (NPSH), ROS and GST activity while γ-GT was unaffected. In the kidney, acute cocaine significantly increased concentration of the whole pool of sulfane sulfur, reduced the content of its bound fraction but H_2_S, sulfate and NPSH levels were unchanged while ROS and activities of GST and γ-GT were reduced. Acute cocaine enhanced activity of the CSE and 3-MST in the liver and kidney, respectively. Repeatedly administered cocaine enhanced the whole pool of sulfane sulfur and reduced H_2_S level simultaneously increasing sulfate content both in the liver and kidney. After repeated cocaine, a significant decrease in ROS was still observed in the liver while in the kidney, despite unchanged ROS content, a marked increase in MDA level was visible. The repeated cocaine decreased 3-MST and increased γ-GT activities in both organs but reduced GST in the kidney. Our results show that cocaine administered at a relatively low dose shifts Cys metabolism towards the formation of sulfane sulfur compounds which possess antioxidant and redox regulatory properties and are a source of H_2_S which can support mitochondrial bioenergetics.

## Introduction

Cocaine (benzoylmethylecgonine), one of the most widespread and addictive drugs of abuse, is a fat-soluble alkaloid derived from the leaves of the South American plant *Erytroxylon coca*. In mammals, cocaine is mainly metabolized by plasma, liver and intestinal carboxylesterases that hydrolyze its ester groups leading to the formation of non-toxic metabolites [1–4]. The minor route of cocaine metabolism catalyzed in the liver by cytochrome P_450_ and flavin-containing monooxygenases involves the amine moiety transformations leading to the formation of oxidative metabolites [3,5–9]. It is believed that toxicity of high cocaine doses is the effect of redox cycling of some reactive metabolites that induces the excessive generation of reactive oxygen species (ROS) causing the oxidative stress and subsequent lipid peroxidation [6–9]. On the other hand, the cocaine-mediated formation of reactive metabolites is restricted primarily to the liver while their low levels in other tissues are unlikely to contribute to the general systemic toxicity of cocaine. Hence, it is postulated that other mechanisms than that evoked by reactive metabolites and ROS can underlie the multiorgan toxicity of cocaine [8,10–12]. It is worth emphasizing that interactions of cocaine with membrane bound proteins [13] and disturbance in the mitochondrial respiratory chain are considered to be significant factors in the acute cocaine toxicity [14–17].

**Fig 1:**
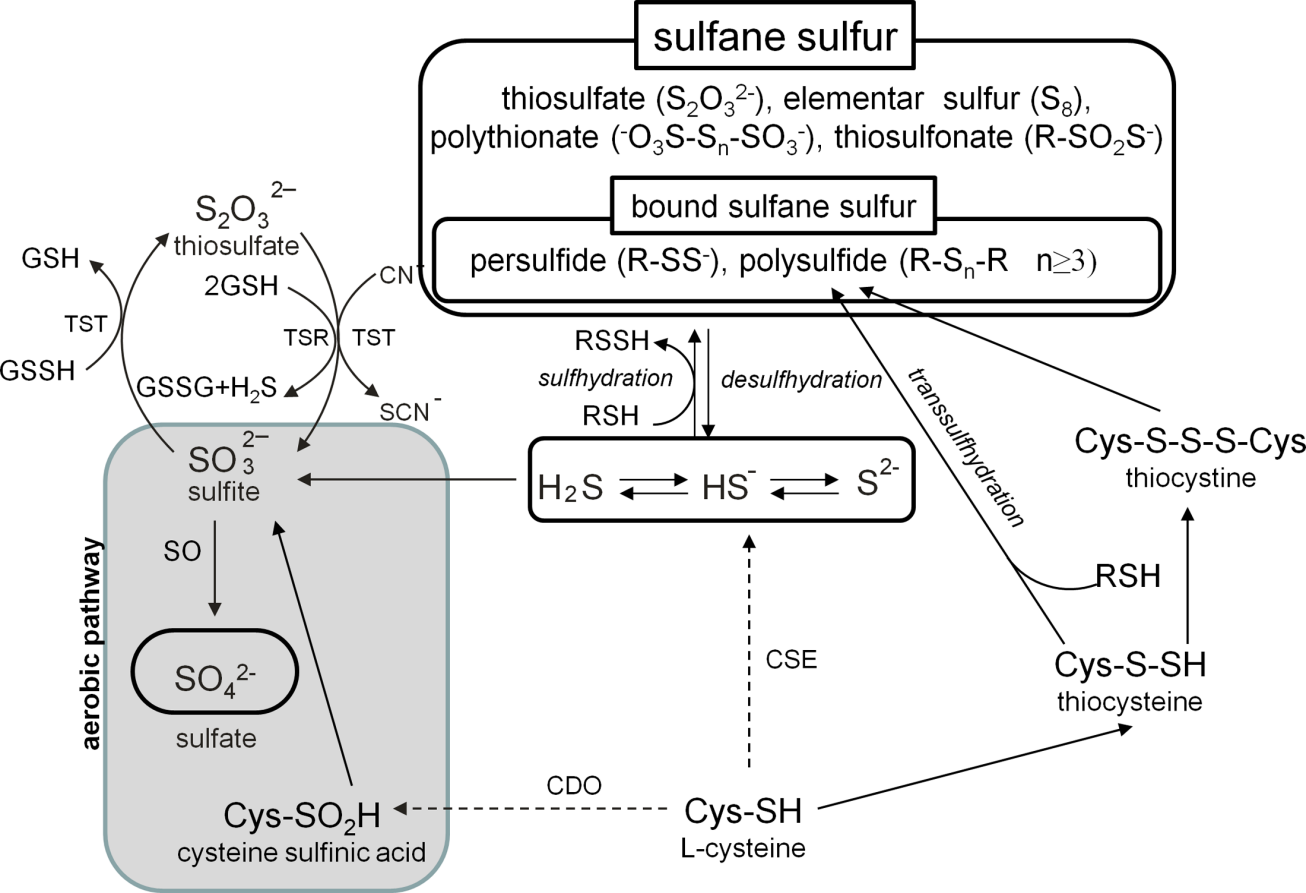
Aerobic and anaerobic metabolism of cysteine. H_2_S formed enzymatically from L- or D-cysteine, in mitochondria is transformed to sulfite which in the reaction catalyzed by SO is oxidized to sulfate (aerobic pathway). Anaerobic metabolism of L-cysteine leads via synthesis of H_2_S and its further sulfhydration to the formation of sulfane sulfur compounds. Abbreviations: CBS—cystathionine β-synthase; CSE—γ-cystathionase; CDO—cysteine dioxygenase; SO—sulfite oxidase; TSR—thiosulfate reductase; TST—rhodanese.

Non-protein (NPSH) and protein thiols, that are a natural reservoir of reductive power of cells and plasma, act as intra- and extracellular thiol redox buffers [18]. The thiol tripeptide GSH and amino acid cysteine (Cys) belong to the most important NPSH in cells. The nucleophilic sulfhydryl (-SH) group of GSH possesses reductive properties and is critical for maintenance of the physiological potential in cells [19]. Cys is not only a rate-limiting amino acid in the GSH biosynthesis but also an important molecule responsible for transduction of redox signaling associated with the formation of sulfane sulfur [20,21]. Cys can be metabolized via an aerobic route producing sulfates (SO_4_^2-^) and taurine in which sulfur atom has the highest (+VI) oxidation state. On the other hand, an anaerobic route of Cys metabolism leads to biosynthesis of compounds containing reduced labile sulfane sulfur always occurring in 0 to -I oxidation state and bound to another sulfur atom [20,21]. Hydropersulfides (R-SSH), polysulfides (R-S_n_R, n ≥ 3) and thiosulfates belong to the most important sulfane sulfur compounds [22] (Fig 1). One of the most abundant R-SSH, thiocysteine (L-cysteine persulfide; CysSSH) is synthesized from cystine in the reaction catalyzed by cystathionine β-synthase (CBS) and cystathionine γ-lyase (γ-cystathionase; CSE) [23] (S1A Fig). The sulfane sulfur atom can be transferred from CysSSH to GSH with the formation of GSH persulfide (GSSH) [23]. R-SSH can be also formed in the process of S-sulfhydration in which hydrogen sulfide anion (HS^-^) reacts with disulfides (RSSR). Compared to thiols, R-SSH are more effective hydrogen donors, and like persulfide anion (RSS^-^), more effective electron donors, what makes them highly effective antioxidants [24]. The sulfane sulfur atom can be transferred from CysSSH or GSSH to a sensor target protein or NPSH with the formation of other R-SSH compounds. The latter reaction called S-transsulfhydration is one of the possible signal transduction pathways associated with the thiol activity (Fig 1). On the other hand, persulfides in reactions with thiols can be biodegraded to hydrogen sulfide (H_2_S) in the process of desulfhydration (Fig 1). H_2_S is mostly metabolized to thiosulfate and sulfate via oxidative metabolism in mitochondria which is triggered by GSH [25–28] (S1B Fig). The mitochondrial sulfide oxidation pathway couples sulfide catabolism to oxidative phosphorylation making sulfide the first known inorganic substrate for the mammalian electron transfer chain [28–32]. Furthermore, a stimulatory effect of low H_2_S concentrations and an inhibitory effect of its high concentrations on mitochondrial bioenergetic functions in the mammalian cells have been recently demonstrated [27,28].

Referring to the impact of cocaine on thiols homeostasis, only a few studies have shown that the administration of its relatively high doses (20–25 mg/kg) significantly increased the GSH and Cys concentrations in the rodent liver [33–36], but such treatment decreased GSH content in plasma [35,37,38]. Moreover, cocaine lowered glutathione peroxidase activity in the liver while it enhanced glutathione reductase activity both in the liver and plasma [35–38]. In contrast, in the rat heart, repeated i.p. or i.v. cocaine administration evoked decreases in the levels of GSH and in glutathione S-transferase (GST) with a simultaneous increase in glutathione disulfide (GSSG) concentration [39]. Also in the primary cultured human proximal tubular epithelial kidney cells, cocaine at low concentrations induced depletion of cellular GSH [40]. Furthermore, in our recently published study, cocaine (10 mg/kg ip) administered acutely increased the total and protein-bound homocysteine (Hcy), decreased sulfane sulfur and did not change different redox forms of Cys concentrations in the rat plasma while given repeatedly increased free Hcy and lowered the total and protein-bound Cys while sulfane sulfur level was unchanged [41]. All the above data indicate that depending on the drug dose, treatment regimen and the studied mammalian tissue, cocaine differently affects low molecular weight thiol concentrations and activities of GSH-dependent enzymes. However, the effects of cocaine on Cys metabolism have not been studied so far. This issue is especially interesting because the products of Cys metabolism such as sulfane sulfur possess antioxidant and redox regulatory properties as well as are a potent source of H_2_S [20,21,27,42]. Thus, they can modulate the level of ROS in the studied tissues, affect redox signaling via the S-sulfhydration and mitochondrial oxidative phosphorylation via oxidation of sulfide to sulfate [20, 28–30,43,44].

In order to clarify the cocaine action via the modulation of Cys homeostasis, we decided to perform a comprehensive comparative analysis of the impact of acute and repeated i.p. cocaine administration on Cys metabolism in the rat liver and kidney and extend our previous observation performed on rat plasma [41]. The liver was chosen as an organ in which the endogenous synthesis and metabolism of Cys occurs most intensively while the kidney as an organ which prevents the loss of Cys from the body. The complex analyses of anaerobic Cys metabolism included determination of its products (i.e. total sulfane sulfur, bound sulfane sulfur, and sulfides as a product of H_2_S dissociation) and activities of enzymes engaged in this metabolism (i.e. CSE, 3-mercaptopyruvate sulfurtransferase (3-MST) and rhodanese (TST) responsible for sulfane sulfur transfer to different acceptors). Aerobic Cys metabolism was assessed on the basis of the level of sulfates. Additionally, the effects of acute and repeated cocaine treatment on the total, non-protein and protein sulfhydryl (-SH) group levels and on the activities of γ-glutamyl-transpeptidase (γ-GT, an enzyme responsible for extracellular degradation of GSH [45]) and glutathione S-transferase (GST, an enzyme responsible for the conjugation of GSH with certain endogenous metabolites or xenobiotics [46]) were measured in these tissues. Furthermore, to evaluate the effect of cocaine on the intensity of oxidative processes, the levels of ROS and malondialdehyde (MDA) were determined as markers of oxidative stress and lipid peroxidation, respectively. Finally, due to the nitric oxide (NO) involvement in the covalent modification of thiols to form the S-nitrosothiols [47], the concentration of stable products of aerobic biodegradation of NO, i.e. nitrates (III) was also assayed. We hope this set of experiments can shed a light on the new mechanism of cocaine action associated with the modulation of sulfur metabolism in mammalian tissues.

## Materials and Methods

### Ethics statement

The experiments were carried out in compliance with the Animal Experiment Bill of January 21, 2005 (published in Journal of Laws no 33/2005 item 289, Poland), and according to the Directive of the European Parliament and of the Council of Europe 2010/63/EU of 22 September 2010 on the protection of animals used for scientific purposes. They received also an approval of the Local Ethics Committee at the Institute of Pharmacology Polish Academy of Sciences (Permit Number 1030/2013 issued: April, 2013). All efforts were made to minimize the number and suffering of animals used.

### Animals

The experiments were conducted on 3 months old male Wistar rats (Charles River, Sulzfeld, Germany) of an initial body weight between 280–320 g kept under standard laboratory conditions; 8 animals per a large cage, at room temperature (22°C) under an artificial light/dark cycle (12/12 h; lights on from 7 am, lights off from 7 pm), with free access to standard laboratory chow and tape water. The animals were experimentally naive.

### Chemicals

Cocaine hydrochloride, 1-chloro-2,4-dinitrobenzene (CDNB), 2,7-dichlorofluorescein (DCFH), 2,7-dichlorofluorescein diacetate (DCFH-DA), dithiothreitol (DTT), 5,5’-dithio-bis-2-nitrobenzoic acid (DTNB), glycine-glycine (Gly-Gly), glutathione reduced form (GSH), lactic dehydrogenase (LDH), L-glutamyl-3-carboxy-4-nitroanilide, 3-mercaptopyruvate (3-MP), 3-methyl-2-benzothiazolinone hydrazine hydrochloride monohydrate (MBTH), NADPH, N-(1-naphthyl)-ethylene-diamine hydrochloride, N-ethylmaleimide (NEM), β-nicotinamide adenine dinucleotide reduced form (NADH), 2-nitro-5-thiobenzoic acid (TNB), *p*-phenylenediamine, potassium cyanide (KCN), potassium thiocyanate (KSCN), pyridoxal 5’-phosphate monohydrate (PLP), pyruvate, sodium hydrosulfide (NaHS), sodium salt L-homoserine, sodium nitrate (III), sodium thiosulfate (Na_2_S_2_O_3_), sulfanilamide, 1,1’,3,3’-tetraethoxypropane (TEP), tetrachloroacetic acid (TCA), thiobarbituric acid (TBA), thionine, zinc acetate, zinc sulfide (ZnS) were provided by Sigma-Aldrich Chemical Company, (St. Louis, USA). Ammonia (NH_3_), barium chloride (BaCl_2_), formaldehyde, ferric chloride (FeCl_3_), hydrochloric acid (HCl), magnesium chloride (MgCl_2_), potassium dihydrogen phosphate (KH_2_PO_4_), perchloric acid (HClO_4_), sodium carbonate (Na_2_CO_3_), sodium hydroxide (NaOH), sodium sulfite (Na_2_SO_3_), thiosulfate and all the other reagents were obtained from the Polish Chemical Reagent Company (P.O.Ch, Gliwice, Poland).

### Drug treatment

Cocaine hydrochloride dissolved in a sterile 0.9% NaCl was given i.p. at a dose of 10 mg/kg, acutely (single dose) or repeatedly for successive 5 days. Control rats received vehicle in the same way. Rats were killed 1 h after single or the last cocaine/vehicle injection, their livers and kidneys were dissected and immediately frozen on dry ice. Then the tissues were stored at -80°C and further procedures were performed within one month.

### Preparation of tissue homogenates

Tissue homogenates assigned for the determination of the studied parameters were prepared in the same way. To prevent undesirable changes in the examined tissues that could happen *ex vivo*, all experimental procedures related with the preparation of tissue homogenates were carried out extremely fast at 4°C. The frozen tissue samples of liver and kidney were weighted and immediately homogenized (1 g of the tissue in 4 ml of 0.1M phosphate buffer, pH 7.4) with a speed 25 000 revolutions per minute (with 15 sec breaks) using IKA-ULTRATURRAX T8 homogenizer.

### Determination of ROS level

ROS were assayed according to the method of Bondy and Guo [48], using DCFH-DA which is deesterified in tissue homogenates to 2’,7’-dichlorofluorescein acid and then oxidized by ROS to fluorescent 2’,7’-dichlorofluorescein (DCF).

Briefly, to 10 μl of homogenate 990 μl of 0.1M phosphate buffer (pH 7.4) and 10 μl of 1.25M DCFH-DA dissolved in ethanol were added. The reaction mixture was incubated at 37°C for 30 min, protecting the samples from light. The measurements were conducted using a fluorometer at wavelengths: A_ex_ = 488 nm and A_em_ = 525 nm. ROS were evaluated using a standard curve for 10μM DCF, and were expressed in DCF μmoles per g tissue.

### Determination of MDA

The level of MDA as a measure of lipid peroxidation was determined using TBA spectrophotometric assay with TEP as a standard [49]. TBA reacts with some products of lipid peroxidation in acidic environment at increased temperature to form a pink compound.

Briefly, to 250 μl of homogenate, 500 μl of 15% TCA and 500 μl of 0.37% TBA were added. The samples were incubated at 100°C for 10 min, cooled and centrifuged at 10 000 x g for 10 min, and absorbance was measured at a wavelength λ = 535 nm. MDA was evaluated using a standard curve for 2.5μM TEP and was expressed in μmoles of TBA per g tissue.

### Determination of the whole pool of sulfane sulfur

Sulfane sulfur level was determined by the method of Wood [50] which is based on the reaction of cyanolysis. It consists in a nucleophilic attack of cyanide on sulfane sulfur-containing compounds: persulfides, polysulfides, thiosulfate, thiosulfonates, polythionates. These compounds react with cyanide in alkaline solution at a temperature of 10–25°C. The product of this reaction thiocyanate (rhodanate) reacts with ferric ions (Fe^3+^) yielding ferric thiocyanate which is red in color. Formaldehyde stabilizes the complex by the reaction with cyanide excess.

Briefly, 80 μl of 1M NH_3_, 720 μl of distilled water and 100 μl of 0.5M KCN were added to 100 μl of homogenate, and mixed thoroughly. The samples prepared in this way were incubated at room temperature for 45 min and 20 μl of 38% formaldehyde solution and 200 μl of the Goldstein reagent containing Fe^3+^cation were added. The precipitate formed in this reaction was centrifuged at 10 000 x g for 10 min. The supernatant was carefully collected and its absorbance was measured at a wavelength λ = 460 nm. The whole pool of sulfane sulfur was evaluated from a standard curve for 1mM KSCN and was expressed in nmoles of KSCN per g tissue.

### Determination of bound sulfane sulfur

Determination of bound sulfane sulfur was based on the modified method of Ogasawara et al. [51]. According to that author the so-called bound sulfur constitutes the pool of sulfur, which is released during reduction by DTT. Sulfide ions produced in the reaction react with *p*-phenylenediamine in the presence of FeCl_3_ yielding a fluorescence dye thionine.

Briefly, to 125 μl of homogenate, 50 μl of borate buffer (pH = 9.0) and 250 μl of 20mM DTT were added. The mixture was incubated at 37°C for 10 min and then 10 μl of 0.1M NaOH, 400 μl of 12.5mM *p*-phenylenediamine and 100 μl of 40mM FeCl_3_ in 6M HCl were added. This reaction mixture was again incubated for 10 min at 37°C. Then, the samples were centrifuged at 13 400 x g at room temperature for 5 min and fluorescence of the mixture was measured at wavelengths: A_ex_ = 600 nm and A_em_ = 623 nm. The bound sulfane sulfur was evaluated from a standard curve for 10μM and 100μM NaHS and was expressed in nmoles of NaHS per g tissue.

### Determination of H_2_S level

H_2_S level was determined using a modification of the method of Tamizhselvi et al. [52]. In alkaline solution, equilibrium of H_2_S dissociation was shifted towards S^2-^ formation. The latter form of H_2_S reacts in the examined tissue homogenates with zinc acetate to form ZnS which then reacts with *p*-phenylenediamine yielding a fluorescent dye (thionine) in the presence of FeCl_3_. Aliquots of homogenate (125 μl) were mixed with 1mM Na_2_CO_3_ (125 μl) followed by incubation at room temperature (30 min). Next, *p*-phenylenediamine (12.5mM), 40mM FeCl_3_ in 6M HCl, and H_2_O (125 μl) were added. After a 10-min incubation at room temperature, the samples were centrifuged at 10 000 x g for 5 min, and fluorescence of the mixture was measured (A_ex_ = 600 nm and A_em_ = 623 nm). H_2_S concentrations were read from a calibration curve prepared from thionine (2.5–25 μM) and were expressed in nmoles of thionine per g tissue.

### Determination of CSE activity

CSE activity was determined by the method of Matsuo and Greenberg [53] with some modifications [54]. L-homoserine was used as a substrate while PLP fulfilled the function of a coenzyme. α-Ketobutyric acid formed from L-homoserine was determined with the use of MBTH.

Briefly, to 50 μl of 1mM PLP, 50 μl of 0.2M of L-homoserine and 650 μl of homogenate diluted 50x (liver) or 5x (kidney) with 0.1M phosphate buffer (pH = 7.4). This reaction mixture was incubated at 37°C for 30 min and then 250 μl of 50% TCA were added. The precipitate was centrifuged at 12 000 x g for 10 min. After centrifugation, 500 μl of the supernatant, 1 ml of 1M acetate buffer (pH = 5.0) and 400 μl of 0.1% MBTH were mixed, incubated at 50°C for 30 min, and cooled. Absorbance of the mixture was measured at a wavelength λ = 320 nm and amount of the product formed was calculated from a standard curve prepared from 2mM α-ketobutyric acid. The activity of the enzyme was expressed as μmoles of product formed during 1 min per mg protein.

## Determination of 3-MST activity

3-MST activity was determined by the method of Valentine and Frankenfeld [55]. The determination has two steps. In the first step, sulfur from 3-MP is transferred by 3-MST yielding pyruvate and thiosulfate. In the next step, pyruvate is reduced to lactate with the participation of LDH and NADH. This method utilizes the difference in absorption spectrum between NADH and NAD^+^ at a wavelength λ = 340 nm. The difference between initial absorbance and its value after LDH addition corresponds to the amount of the pyruvate formed.

Briefly, the reaction mixture containing: 250 μl of 0.12M phosphate buffer (pH = 8.0), 50 μl of 0.5M Na_2_SO_3_, 50 μl of 0.15M DTT, 50 μl of distilled water, 50 μl of homogenate diluted 100x with 0.1M phosphate buffer (pH = 7.4) and 50 μl of 0.1M 3-MP was incubated at 37°C for 15 min. Subsequently, 250 μl of 1.2M HClO_4_ were added and the mixture was centrifuged at 3 000 x g for 10 min. After centrifugation, 100 μl of the supernatant were collected and supplemented with 1.2 ml of 0.12M phosphate buffer (pH 8.0) and 100 μl of 0.1M NEM and 50 μl of NADH. Then, 5 μl of LDH were added and absorbance was measured 1 min after LDH addition. Absorbance was measured at a wavelength λ = 340 nm before and after LDH addition and the amount of the pyruvate formed was calculated from a standard curve prepared from 1mM pyruvate. The activity of the enzyme was expressed as μmoles of product formed during 1 min per mg protein.

### Determination of TST activity

TST activity was determined as described previously [56]. The reaction involves transfer of the outer sulfur atom of thiosulfate (sulfane sulfur atom) to cyanide catalyzed by TST with the formation of thiocyanate (rhodanate) and sulfate (IV). The amount of rhodanate is measured colorimetrically using the color reaction of thiosulfate with ferric ions Fe^3+^.

Briefly, reaction mixture containing: 100 μl of homogenate diluted 100 x with 0.1M phosphate buffer (pH 7.4), 100 μl of distilled water, 125 μl of 0.25M Na_2_S_2_O_3_, 125 μl of 0.2M KH_2_PO_4_ and 75 μl of 0.5M KCN was incubated at room temperature for 5 min. Then, 125 μl of 38% formaldehyde solution and 600 μl of the Goldstein reagent were added. The mixture was centrifuged and the supernatant was carefully collected. Absorbance was measured at a wavelength λ = 460 nm and the amount of the product formed was calculated from a standard curve prepared from 2,5mM KSCN. The activity of the enzyme was expressed as μmoles of product formed during 1 min per mg protein.

### Determination of inorganic sulfates concentration

The method is based on the precipitation reaction of barium sulfate in gelatin solution that stabilizes turbidity [57].

Briefly, gelatin solution was prepared on the day preceding determination and was left at +4°C. Two hours before the assay, 0.1 g of BaCl_2_ was dissolved in that solution. Homogenate was deproteinized by the addition of 50 μl of 50% TCA to 1 ml and the mixture was centrifuged at 10 000 x g. To 300 μl of supernatant 200 μl of distilled water and 500 μl of BaCl_2_ solution in gelatin were added in sequence. After 20 min, turbidity was measured against a blank sample. A standard curve was prepared from 5mM sodium sulfate (VI) solution. Sulfate concentrations were read from a calibration curve prepared from 5mM sodium sulfate (VI) solution and was expressed in nmoles per g tissue.

### Determination of the total-, non-protein and protein -SH group levels

Determination of the total and NPSH levels is based on the Ellman's method in which DTNB is reduced by–SH group to TNB characterized by intensive yellow color, which shows maximum absorbance at 412 nm [58].

For determination of the total -SH group level, the reaction mixture composed of 25 μl of the examined tissue homogenate, 175 μl of 0.2M buffer Tris-HCl + 0.02M EDTA (pH 8.2), 10 μl of DTNB in methanol and 790 μl of methanol, was incubated for 15 min at room temperature and then the sample was centrifuged at 10 000 x g for 10 min. The level of total–SH groups was determined from a standard curve prepared for the 1mM GSH and was expressed in μmoles per g tissue.

For determination of NPSH level, 950 μl of the studied homogenate was first deproteinized by addition of 50 μl of cold 50% TCA, and then the sample was centrifuged at 10 000 x g at a temperature of +4°C for 10 min. To 850 μl of 0.2M phosphate buffer (pH 8.2), 100 μl of 6mM DTNB and 50 μl of supernatant from deproteinized homogenate were added. Absorbance was measured at a wavelength λ = 412 nm 1 min after supernatant addition. The total content of NPSH was determined from a standard curve prepared for the 1mM GSH and was expressed in μmoles per g tissue.

The protein -SH group level for each sample was calculated as a difference between the total–SH and NPSH group levels.

### Determination of γ-GT activity

γ-GT activity was measured by the method of Orłowski and Meister [59] consisting in enzymatic transformation of L-glutamyl-3-carboxy-4-nitroanilide to colored p-nitroaniline, which shows maximum absorbance at 410 nm.

Briefly, the reaction mixture composed of 475 μl of the solution containing 5.5mM L-glutamyl-3-carboxy-4-nitroanilide, 11mM MgCl_2_ and 22.2mM Gly-Gly was incubated with 25 μl of homogenate at a temperature of 37°C for 30 min. The reaction was stopped by the addition of 1 ml of 1.5M acetic acid and then the samples were centrifuged at 10 000 x g for 5 min. The absorbance of p-nitroaniline formed during 5 min of incubation was measured at a wavelength λ = 410 nm and amount of the formed product was calculated from a standard curve prepared from 1mM p-nitroaniline. The activity of the enzyme was expressed as μmoles of product formed during 1 min per mg protein.

### Determination of GST activity

GST activity was measured by the method of Habig et al. [60]. GST catalyzed reaction between GSH and CDNB resulted in formation of 2,4-dinitrophenyl-S-glutathione which has maximum absorbance at a wavelength λ = 340 nm.

Briefly, 50 μl of 20mM solution of GSH, 50 μl of 20mM solution of CDNB and 50 μl of liver or kidney homogenate diluted 50x with 0.1M phosphate buffer pH 7.4 were added to 850 μl of 0.1M phosphate buffer pH 6.5. Absorbance was measured 15 and 75 sec latter at a wavelength λ = 340 nm. Enzyme activity was determined as a difference between an increase in the absorbance of the tested and control samples divided by the value of millimolar absorbance coefficient for the formed conjugate. The activity of the enzyme was expressed as μmoles of the product formed during 1 min per mg protein.

### Determination of NO level

The method is based on the assumption that all nitrates (III) derive from NO. Nitrates (III) react with sulfanilamide and N-(1-naphthyl)-ethylene-diamine hydrochloride to form red-violet diazo dye showing absorbance in visible range [61].

In order to inactivate NO synthase, the sample containing 250 μl of homogenate and 750 μl of double distilled water was heated in a boiling water bath for 15 min. To microplate wells, 75 μl of supernatant and 75 μl of double distilled water were added and incubated at room temperature for 30 min. After incubation, absorbance was read at a wavelength λ = 540 nm. In order to obtain a colored product, 50 μl of 1% sulfanilamide in 2.5% phosphoric acid and 50 μl of 0.1% N-(1-naphthyl)-ethylenediamine hydrochloride in 2.5% phosphoric acid were added to microplate wells. The mixture was incubated at room temperature in the dark for 15 min and absorbance was read again at 540 nm. NO content was calculated based on a standard curve prepared from 10 μM sodium nitrate (III) and was expressed in μmoles per g tissue.

### Determination of protein concentration

The determination of protein concentration was carried out according to the method described by Lowry et al. [62].

### Statistics

The significance of differences in the examined parameters in the rat liver and kidney between the control group and that receiving i.p. cocaine (acutely or repeatedly) was estimated by Student's t-test. The *p* values of less than or equal to 0.05 were considered to indicate statistical significance. The statistical analysis was done using STATISTICA 10.0 Software (Statsoft, Inc, USA).

## Results

### Effects of acute and repeated cocaine administration on ROS and MDA as well as on -SH group levels in the rat liver and kidney

**Fig 2:**
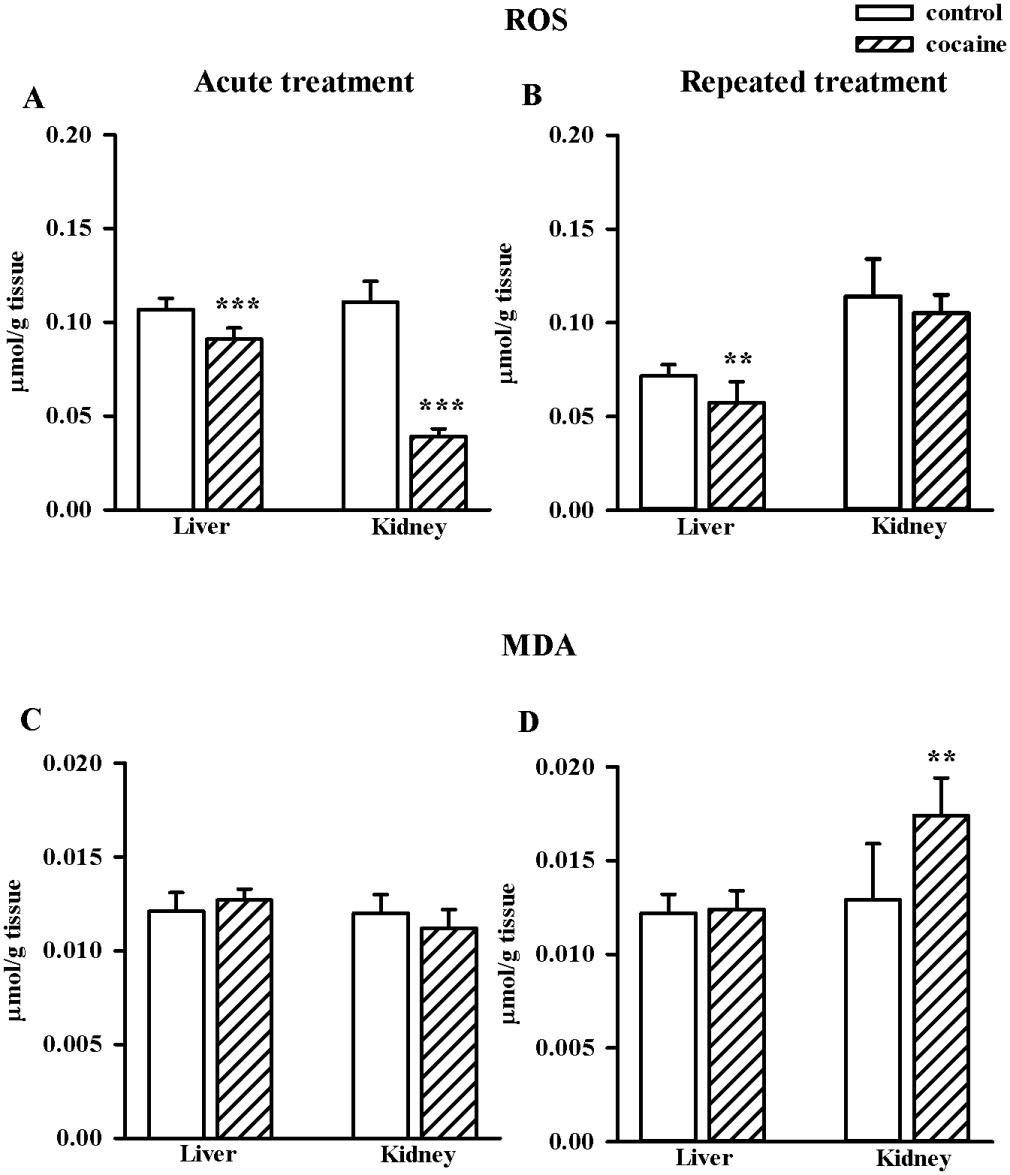
Effects of acute and repeated cocaine (10 mg/kg i.p.) treatment on the levels of ROS (2A, 2B) and lipid peroxidation products (MDA; 2C, 2D) in the rat liver and kidney. Data are presented as the mean ± SD, n = 8–10/group, **p < 0.01, ***p < 0.001 vs. corresponding control group (Student's t-test).

One hour after acute cocaine administration significant decreases in the tissue concentrations of ROS were observed both in the liver (n = 8/group, t = 5.332, df = 14, *p* < 0.001) and kidney (n = 8/group, t = 16.906, df = 14, *p* < 0.001), but 1 h after the repeated drug treatment this effect was still visible only in the liver (n = 10/group, t = 3.642, df = 18, *p* < 0.01) (Fig 2). In both organs there were no changes in the lipid peroxidation measured as the levels of MDA products after acute cocaine treatment. However, the repeated cocaine administration increased MDA content in the rat kidney (n = 10/group, t = -3.992, df = 18, *p* < 0.01) (Fig 2).

**Fig 3:**
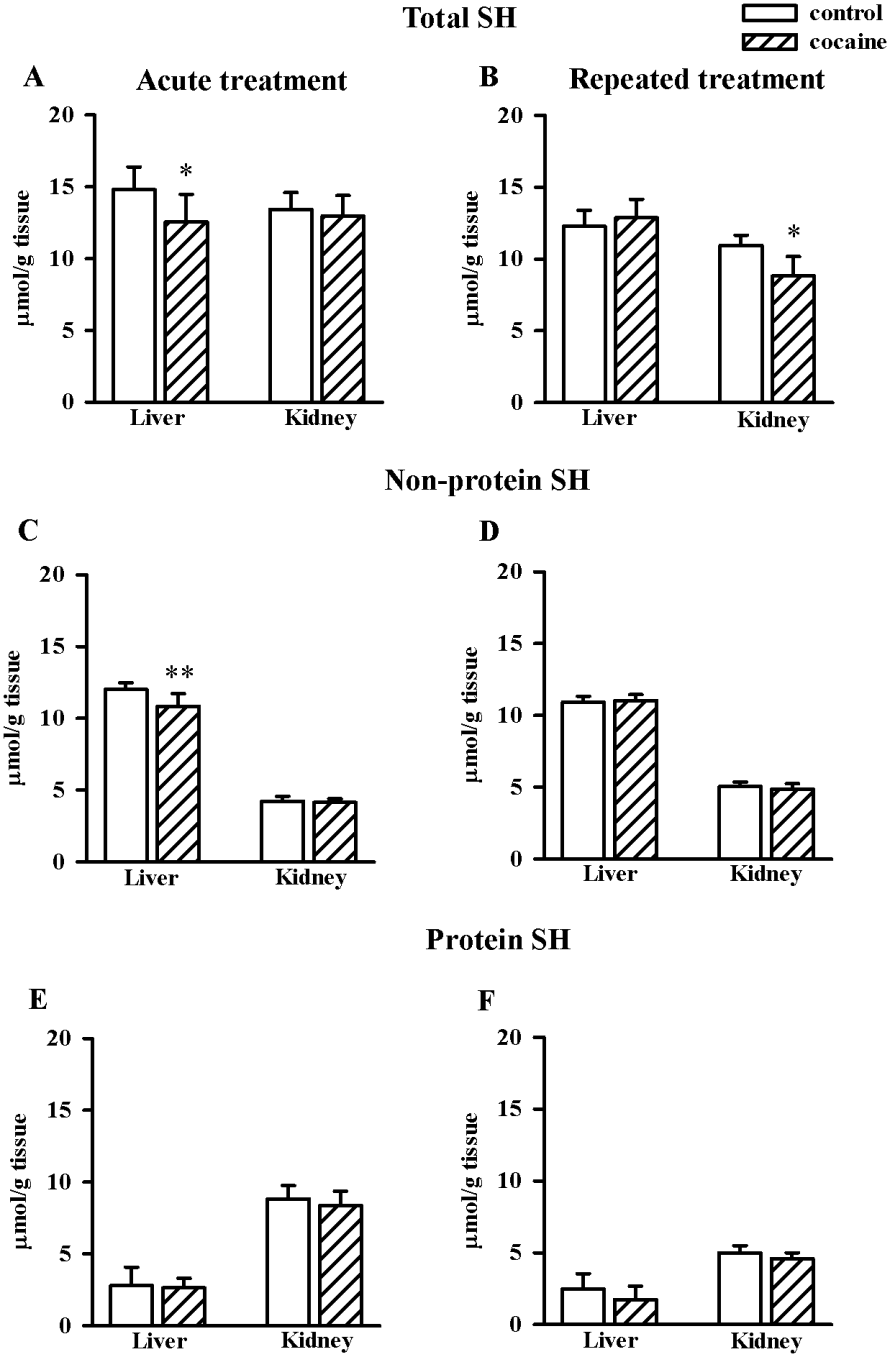
Effects of acute and repeated cocaine (10 mg/kg i.p.) treatment on the total-, non-protein- and protein -SH group levels in the rat liver and kidney (A-F). Data are presented as the mean ± SD, n = 8–10/group, *p < 0.05, **p < 0.01 vs. corresponding control group (Student's t-test).

In the rat liver, after acute (but not repeated) cocaine administration, the concentrations of the total (n = 8/group, t = 2.567, df = 14, *p* < 0.02) and non-protein -SH (n = 8/group, t = 3.469, df = 14, *p* < 0.01) group levels were decreased markedly compared to the appropriate controls (Fig 3). In contrast to the liver, in the kidney only repeated cocaine treatment induced a significant decrease in the total -SH group content (n = 10/group, t = 2.527, df = 18, *p* < 0.02) (Fig 3). There were no changes in protein -SH group levels in the liver and kidney both after acute and repeated cocaine administration (Fig 3).

### Effects of acute and repeated cocaine administration on γ-GT and GST activities in the rat liver and kidney

**Fig 4:**
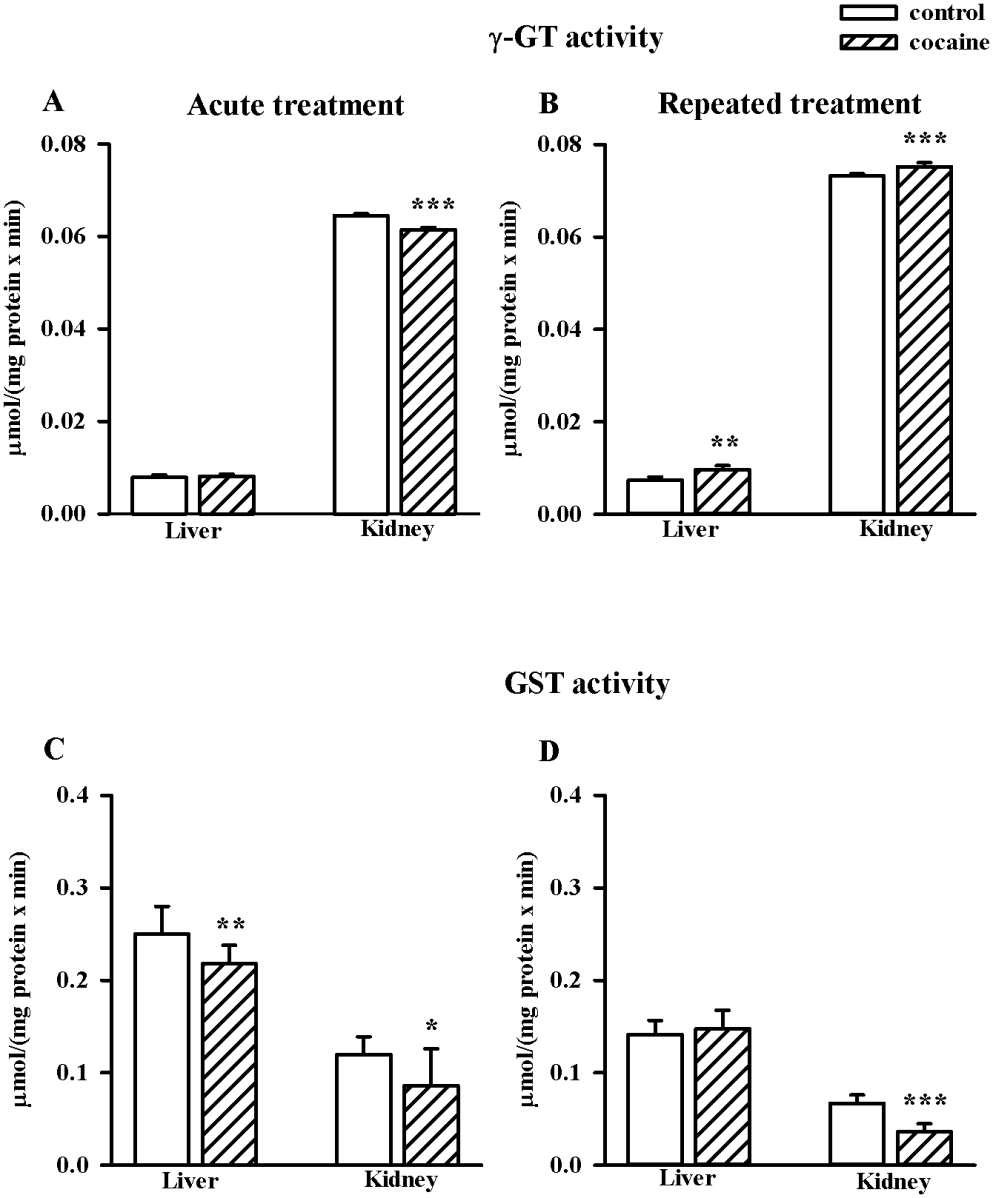
Effects of acute and repeated cocaine (10 mg/kg i.p.) treatment on enzymatic activities of γ-GT (A and B) and GST (C and D) in the liver and kidney. Data were presented as the mean ± SD, n = 8–10/group, *p < 0.05, **p < 0.01, ***p < 0.001 vs. corresponding control group (Student's t-test).

An acute cocaine injection decreased γ-GT activity only in the kidney (n = 8/group, t = 13.228, df = 14, *p* < 0.001), while its repeated administration increased its activity both in the liver (n = 10/group, t = -3.458, df = 18, *p* < 0.01) and kidney (n = 10/group, t = -6.240, df = 18, *p* < 0.001) (Fig 4). The enzymatic activity of GST after a single cocaine dose significantly decreased both in the liver (n = 8/group, t = 2.973, df = 14, *p* < 0.01) and kidney (n = 8/group, t = 2.155, df = 14, *p* < 0.05) while after the last repeated cocaine dose only in the kidney (n = 10/group, t = 7.711, df = 18, *p* < 0.001) (Fig 4).

### Effects of acute and repeated cocaine administration on the concentrations of sulfane sulfur, bound sulfane sulfur, H_2_S and sulfates in the rat liver and kidney

**Fig 5:**
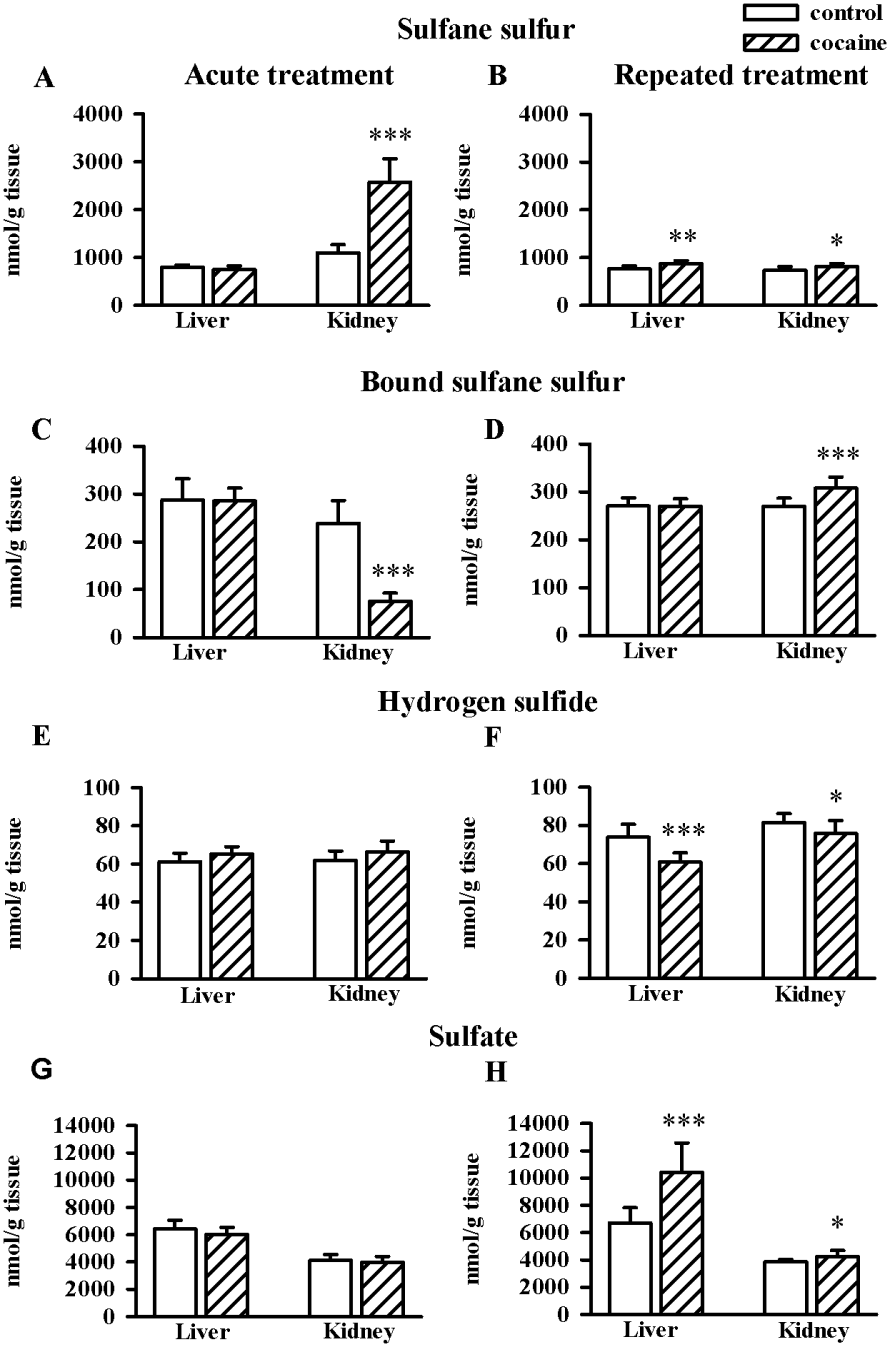
Effects of acute and repeated cocaine (10 mg/kg i.p.) treatment on the levels of sulfane sulfur (A and B), bound sulfane sulfur (C and D), hydrogen sulfide (E and F) and sulfate (G and H) in the rat liver and kidney. Data are presented as the mean ± SD, n = 8–10/group, *p < 0.05, **p < 0.01, ***p < 0.001 vs. corresponding control group (Student's t-test).

In the rat kidney, acute cocaine administration significantly increased the whole pool of sulfane sulfur (n = 8/group, t = -8.046, df = 14, *p* < 0.01) and simultaneously decreased bound sulfane sulfur level (n = 8/group, t = 9.088, df = 14, *p* < 0.001), but such changes were not visible in the liver (Fig 5). Repeated cocaine treatment enhanced the content of the whole pool of sulfane sulfur in both organs (n = 10/group, liver, t = -3.637, df = 18, *p* < 0.002 kidney, t = -2.464, df = 18, *p* < 0.05) while bound sulfane sulfur was raised only in the kidney (n = 10/group, t = -4.125, df = 18, *p* < 0.001). Acute cocaine treatment changed neither H_2_S nor sulfate levels in the rat liver and kidney. However, its repeated administration decreased H_2_S concentrations (n = 10/group, liver, t = 5.005, df = 18, *p* < 0.001; kidney, t = 2.174, df = 18, *p* < 0.05) while increasing sulfate contents in both these organs (n = 10/group, liver, t = -4.775, df = 18, *p* < 0.01; kidney, t = -2.321, df = 18, *p* < 0.05) (Fig 5).

### Effects of acute and repeated cocaine treatment on enzymatic activities of CSE, 3-MST and TST in the rat liver and kidney

**Fig 6:**
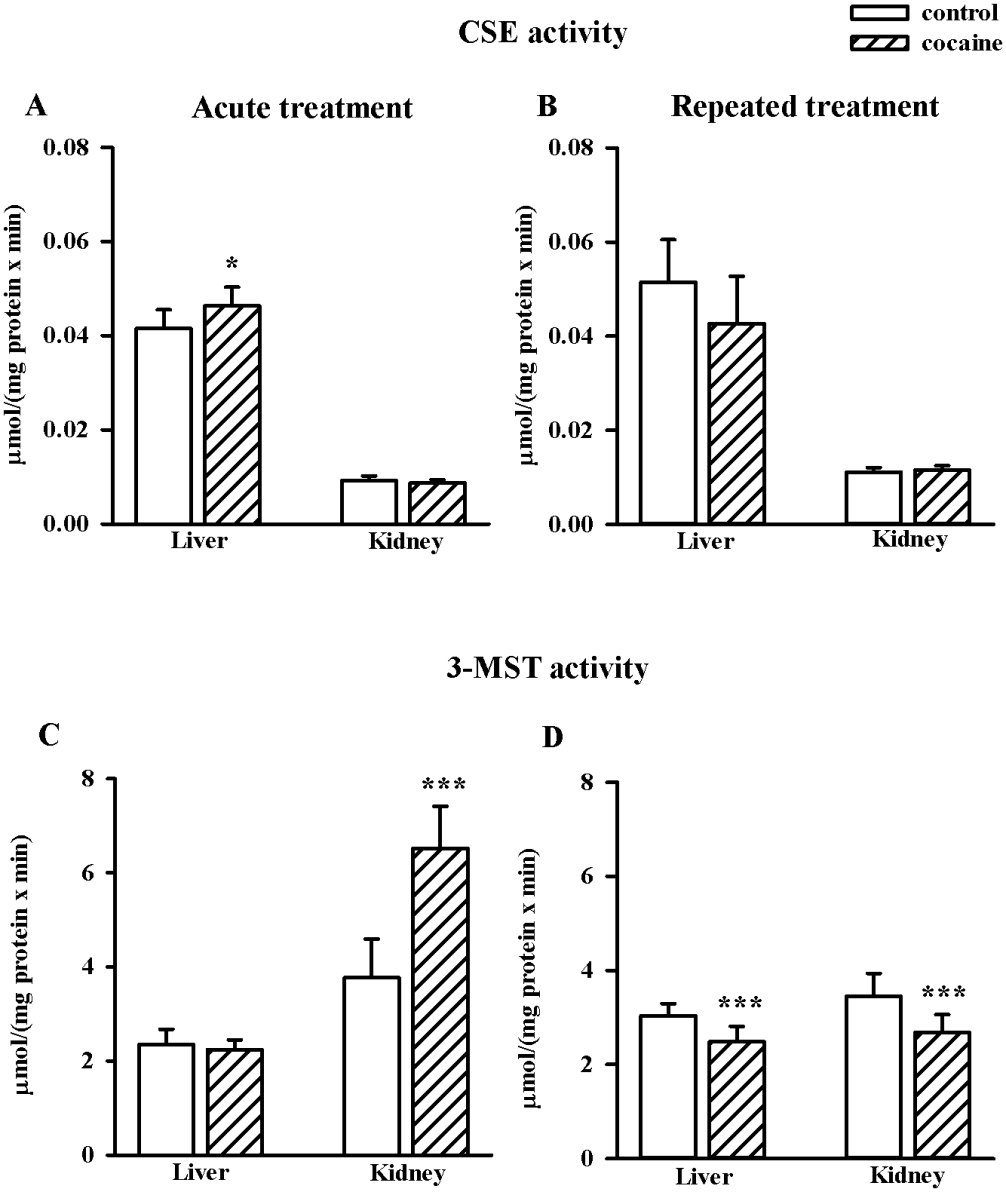
Effects of acute and repeated cocaine (10 mg/kg i.p.) treatment on activities of CSE (A and B) and 3-MST (C and D), enzymes which are involved in the anaerobic Cys metabolism in the rat liver and kidney. Data are presented as the mean ± SD, n = 8–10/group, *p < 0.05, ***p < 0.001 vs. corresponding control group (Student's t-test).

In general, enzymatic activity of CSE was much higher in the liver than in the kidney, in contrast to 3-MST activity which was much higher in the kidney than in the liver. Acute cocaine administration markedly enhanced CSE activity in the liver (n = 8/group, t = -2.526, df = 14, *p* < 0.05) while 3-MST in the kidney (n = 8/group, t = -6.429, df = 14, *p* < 0.001) (Fig 6). However, its repeated treatment significantly decreased 3-MST activity in both organs (n = 10/group, liver, t = 4.198, df = 18, *p* < 0.001; kidney, t = 3.930, df = 18, *p* < 0.001) and did not change CSE activities (Fig 6). TST activity was not changed in the liver and kidney by both acute and repeated cocaine treatment (data not shown).

### Effects of acute and repeated cocaine treatment on NO levels in the rat liver and kidney

**Fig 7:**
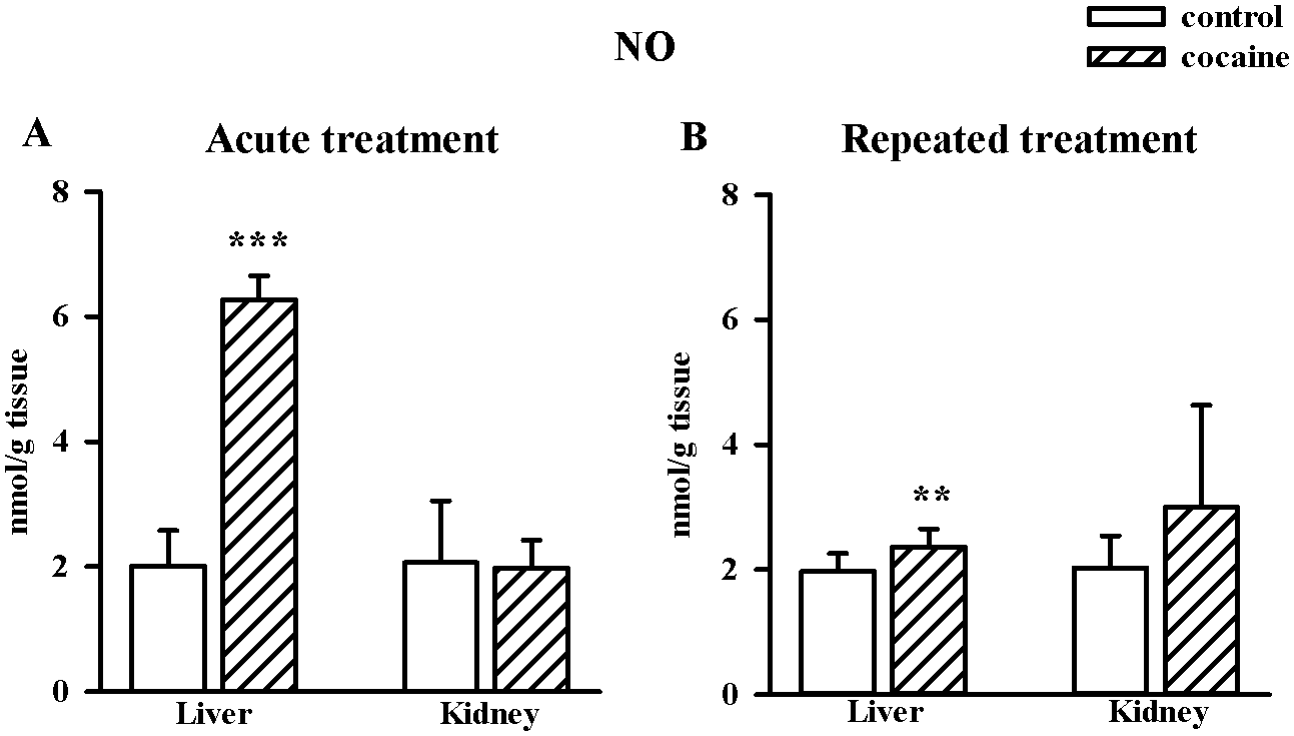
Effects of acute and repeated cocaine (10 mg/kg i.p.) treatment on the concentration of NO measured as the level of its stable metabolite nitrate (III) in the rat liver and kidney. Data are presented as the mean ± SD, n = 8–10, **p < 0.01, ***p < 0.001 vs. corresponding control group (Student's t-test).

Acute and repeated cocaine treatment led to the increase in NO level in the rat liver (acute, n = 8/group, t = -17.660, df = 14, *p* < 0.001; repeated, n = 10/group, t = -3.053, df = 18, *p* < 0.01), but no changes were visible in the rat kidney (Fig 7).

## Discussion

The impact of cocaine on the metabolism of sulfur-containing compounds in the peripheral tissues is poorly understood. In the present study, for the first time we simultaneously assessed changes in the anaerobic and aerobic Cys metabolism and in the oxidative processes in the liver and kidney of rats treated with this drug of abuse.

Our results indicate that in the tested tissues acute and repeated administration of cocaine affects thiol homeostasis and oxidative processes in a different way. It is commonly accepted that cocaine evokes tissue damage due to the generation of ROS [6,8,12]. However, in our study, unexpectedly, this drug of abuse used at a dose of 10 mg/kg markedly decreased ROS concentrations in the rat liver both after acute and repeated treatment while in the kidney only after acute injection. The ROS scavenging is closely linked with the cellular thiol concentrations [63, 64]. Consistently, in the rat liver 1 hour after the administration of a single dose of cocaine, a significant decline in the NPSH level which, in fact, reflects GSH concentration could explain the observed decrease in ROS content. However, in the rat kidney in which a decrease in the ROS content was much greater than in the liver at that time point, the reduction in NPSH content was not observed. Furthermore, in the liver after repeated cocaine administration, the persisting decrease in the ROS content was no longer accompanied by a decline in the NPSH level. Hence, the above data seem to indicate that reduction in the ROS concentration was not associated with their scavenging by GSH [63,64], but with antioxidant properties of sulfane sulfur [20,23] the content of which increased especially in the rat kidney after acute cocaine administration and in both organs, although to a lesser extent, after its repeated injections. However, the most intriguing effect after repeated cocaine treatment was observed in the rat kidney, in which despite the lack of statistically significant enhancement in the concentration of ROS, a significant increase in the level of MDA was found. The latter effect clearly suggests that the cocaine-induced lipid peroxidation may occur independently of the enhanced ROS production.

Recently researchers' attention has been focused on another group of reactive molecules which are defined as "reactive sulfur species" (RSS) [65–69]. Although RSS production was initially suggested to be primarily due to oxidative stress [65,66], many of these molecules exist independently of it in the cell or are synthesized under non-oxidative conditions. RSS are either byproducts of the reactions of major thiol-containing molecules, such as GSH, Cys and dithiols (e.g. lipoate) or a result of oxidation of sulfite or sulfate molecules. It has been demonstrated in liposomal and skeletal muscle model systems that sulfite radicals are able to initiate lipid oxidation [67]. So it is likely that also in the kidney of rats treated repeatedly with cocaine, sulfite radicals could evoke lipid oxidation. However, further studies are necessary to confirm this assumption.

As to Cys metabolism in the rat liver after acute cocaine administration, there were no changes in the concentrations of the whole pool of sulfane sulfur, its bound fraction, H_2_S and sulfate. However, the increased activity of CSE that plays a predominant role in H_2_S biosynthesis in the liver, seems to suggest that H_2_S concentration should be enhanced, but unfortunately such an increase in its content was not observed in our study. H_2_S is known to be an extremely toxic substance for aerobic organisms [70]. At high concentrations it inhibits mitochondrial respiration by blocking the complex IV in the mitochondrial electron transport chain while at low concentrations H_2_S serves as an inorganic substrate and electron donor for ATP generation [29,30]. Therefore, to maintain the physiological tissue content of H_2_S both reactions responsible for its synthesis and those contributing to H_2_S rapid clearance have to be strictly regulated [71–73]. Under normal conditions, CSE produces H_2_S from Cys but in the presence of a high concentration of Hcy, the latter amino acid becomes a prevailing substrate in the production of H_2_S (S1A Fig) [71,74,75]. Since in our previous study [41], we have demonstrated that cocaine markedly increased concentrations of different redox forms of Hcy in the rat plasma, it seems that Hcy may be a predominant substrate for H_2_S synthesis in cocaine users.

The effects of acute cocaine administration on Cys metabolism in the rat kidney differ markedly from those described for the liver. In fact, there was a huge increase in the kidney concentration of the whole pool of sulfane sulfur and a drastic decline in the content of bound sulfane sulfur, but there were no changes in the levels of H_2_S or sulfate. In the kidney, in contrast to the liver, activity of 3-MST, other H_2_S synthesizing enzyme distinctly increased. 3-MST produces H_2_S from 3-MP which is synthesized from L-Cys and α-ketoglutarate by Cys aminotransferase (CAT) [76] and additionally from D-Cys via D-amino acid oxidase (DAO), an enzyme that also predominantly occurs in the kidney (S1A Fig) [77]. Moreover, the reduction of bound sulfane sulfur by endogenous reducing factors, such as thioredoxin (Trx) and dihydrolipoic acid (DHLA) can generate a considerable amount of H_2_S [78].

It is well known that in mitochondria H_2_S is oxidized to non-toxic sulfur compounds whose ratio and production rate vary in a tissue-specific manner [79–82]. The intensity of H_2_S oxidation in different tissues is usually monitored in an indirect way by the production of thiosulfate and sulfate or by the oxygen consumption [31,32,81,83]. Consistently, it has been directly demonstrated that during incubation of the chicken liver mitochondria with low concentrations of H_2_S (10–60 μM) molecular oxygen was consumed at an accelerated rate and ATP was produced [83]. In our study, the oxidation of H_2_S in the rat liver and kidney was assessed mainly based on the sulfate concentration while thiosulfate was determined in the whole pool of sulfane sulfur but we did not measure oxygen consumption. In reference to H_2_S metabolism, in the rat kidney there were no visible changes in the sulfate level after acute cocaine administration. However, such treatment significantly increased the concentration of the whole pool of sulfane sulfur suggesting that the formation of thiosulfate was markedly enhanced. As the latter increase was accompanied by a dramatic decrease in the content of the bound sulfane sulfur, it was assumed that H_2_S released from this pool of sulfane sulfur in mitochondria was immediately oxidized to thiosulfate increasing in this way the whole pool of sulfane sulfur.

The above-described disparities in the responses of the liver and kidney to acute cocaine administration appear to be related to the tissue-specific differences in the thiol homeostasis. The liver is an organ that contains high concentration of GSH and a relatively low activity of γ-GT [84]. Furthermore, in the hepatic cells, Cys which is a rate limiting factor in GSH synthesis, can be formed endogenously from methionine, especially in conditions of dietary Cys deficiency [85]. In contrast, the kidney is characterized by a high content of Cys [73], a relatively low concentration of GSH and a very high activity of γ-GT [84], the only enzyme that initiates extracellular catabolism of GSH and is responsible for maintenance of the physiological concentration of Cys in cells and extracellular space. Furthermore, γ-GT catalyzes the degradation of other substrates containing the GSH molecule in their structures, such as S-nitrosoglutathione and glutathione S-conjugates [86]. The high activity of this enzyme in the kidney is particularly important in preventing the loss of Cys from the body and, therefore, this organ is a potent source of this amino acid. Interestingly, acutely administered cocaine markedly decreased γ-GT enzymatic activity in the kidney possibly to prevent the excessive release of Cys from the degraded substrates and its further metabolism that could lead to the overproduction of H_2_S. On the other hand, the maintenance of adequate concentrations of GSH in the kidney is essential for conversion of thiosulfate to sulfate (Fig 1). With regard to the latter reaction, the decreases in the enzymatic activity of both γ-GT and GST help to increase the pool of available GSH. Thus, first, thiosulfate is converted into sulfite by either a GSH-dependent thiosulfate reductase (TSR) or TST [85] and then sulfite is oxidized by the mitochondrial enzyme SO to sulfate (Fig 1) [87]. The involvement of GSH-dependent TSR in the sulfite production from thiosulfate is consistent with previously described dependence of sulfate production by hepatocytes on GSH [88]. The latter study demonstrated that depletion of GSH markedly reduced sulfate formation but increased the accumulation of thiosulfate. Hence, it is not surprising that in the rat kidney, in which GSH concentration is much lower than in the liver despite the decreases in γ-GT and GST enzymatic activities after acute cocaine administration, no increase in the content of sulfate was found but the level of thiosulfate, being included in the whole pool of sulfane sulfur was distinctly enhanced.

In contrast to acute cocaine effect, its repeated administration evoked quite different changes in the anaerobic and aerobic Cys metabolism in the rat liver and kidney. Consistently, cocaine given for 5 days significantly decreased the level of H_2_S and simultaneously increased the concentrations of the whole pool of sulfane sulfur (including thiosulfate) as well as sulfate. Moreover, repeatedly administered cocaine differently affected activities of H_2_S synthesizing enzymes. In both organs, the activity of 3-MST was markedly reduced while CSE was maintained at the control level. It is believed that the decrease in the 3-MST activity mainly affected the mitochondrial fraction of this enzyme and its aim was to control H_2_S synthesis and its subsequent oxidation. Although the degree of 3-MST inhibition was comparable in both organs, the decrease in H_2_S content was more pronounced in the liver than in the kidney. Furthermore, after repeated cocaine administration, in the rat liver, a more intensive H_2_S oxidation to sulfate resulted in much higher concentration of sulfate than thiosulfate determined in the whole pool of sulfane sulfur, and in no changes in the content of bound sulfane sulfur. The latter effects suggest that in the liver the concentration of GSH measured as NPSH was sufficient to effectively metabolize H_2_S to sulfate. In opposite, in the kidney, repeatedly administered cocaine induced the most distinct increase in the concentration of bound sulfane sulfur while increases in the levels of sulfate and the whole pool of sulfane sulfur were relatively small. The latter effects indicate that in the kidney some part of the synthesized H_2_S was stored in the form of bound sulfane sulfur. The latter effect and the decreased GSH level in the rat kidney also implicate that H_2_S metabolism to sulfate was less effective. On the other hand, the cocaine-induced formation of bound sulfane sulfur suggests that the process of S-sufhydration is increased in the rat kidney and may be involved in modulating the function of certain proteins. This assumption implies that S-sulfhydration may be a new form of signal transduction in cocaine users, at least in some tissues.

Summing up, the results of the present study show for the first time that cocaine administered at a moderate dose of 10 mg/kg, acutely or repeatedly, differently affects anaerobic and aerobic metabolism of Cys, thiol homeostasis and oxidative processes in the rat liver and kidney. Specific changes in these organs may have significant implications for biological activity of this drug of abuse. It is hypothesized that a moderate dose of cocaine acting through the modulation of Cys metabolism, H_2_S synthesis and its further transformation leading to sulfate formation may affect mitochondrial bioenergetics and cellular signaling.

## Supporting Information

S1 Fig (A) Pathways of H2S synthesis from L- and D-cysteine (Cys) in the mammalian tissues. (B) A scheme showing the H2S oxidation pathway leading to the formation of thiosulfate and sulfate in mitochondria. (A) H_2_S can be produced by three enzymes i.e., cystathionine β-synthase (CBS), cystathionine γ-lyase (CSE) and 3-mercaptopyruvate sulfur transferase (3-MST). CBS and CSE are pryridoxal-5-phosphate-dependent enzymes localized in the cytosol that in the transsulfuration pathway use L-Cys and homocysteine (Hcy) as substrates for H_2_S production [Kabil and Banerjee 2010; Singh and Banerjee 2011]. Hcy, which is formed from methionine in the methionine cycle, in the reaction catalyzed by CBS produces cystathionine at the same time releasing a H_2_S molecule. Next, CSE converts cystathionine into L-Cys that is spontaneously oxidized to cystine. As a substrate for CSE, L-Cys is further metabolized to L-cysteine persulfide known as thiocysteine from which H_2_S is generated in a non-enzymatic manner. The third enzyme 3-MST, synthesizes H_2_S from 3-mercaptopyruvate (3-MP) which is formed from L-Cys in a reaction catalyzed by cysteine aminotransferase (CAT) or from D-cysteine via D-amino acid oxidase (DAO). 3-MST and CAT are ubiquitous enzymes which are localized both in the mitochondria and cytosol, while the occurrence of DAO is only restricted to the kidney and brain peroxisomes [76]. The 3-MST-catalyzed reaction requires a reducing agent (RSH) to release H_2_S from persulfide (R-SSH) formed from the substrate, 3-MP [Kabil and Banerjee 2010; Nagahara et al. 2007]. (B) In the first step, H_2_S is oxidized by mitochondrial membrane-bound flavoprotein, sulfide quinone oxidoreductase (SQR), which forms a protein-bound persulfide while electrons are transferred to ubiquinone (Q_ox_). In the next steps, a sulfur dioxigenase (ETHE1) oxidizes SQR-bound sulfane sulfur to sulfite (SO_3_^2-^), which is subsequently converted to thiosulfate (S_2_O_3_^2-^) by the transfer of a second persulfide equivalent catalyzed by the rhodanese (TST) [73]. Alternatively, SO_3_^2-^ can be oxidized to sulfate (SO_4_^2-^) by sulfite oxidase (SO).
(TIF)
