# Supplementary figures and images for: Cysteine Metabolism and Oxidative Processes in the Rat Liver and Kidney after Acute and Repeated Cocaine Treatment
Source: PLoS One. 2016 Jan 25;11(1):e0147238. doi: 10.1371/journal.pone.0147238 (PMC4726505; doi:10.1371/journal.pone.0147238)

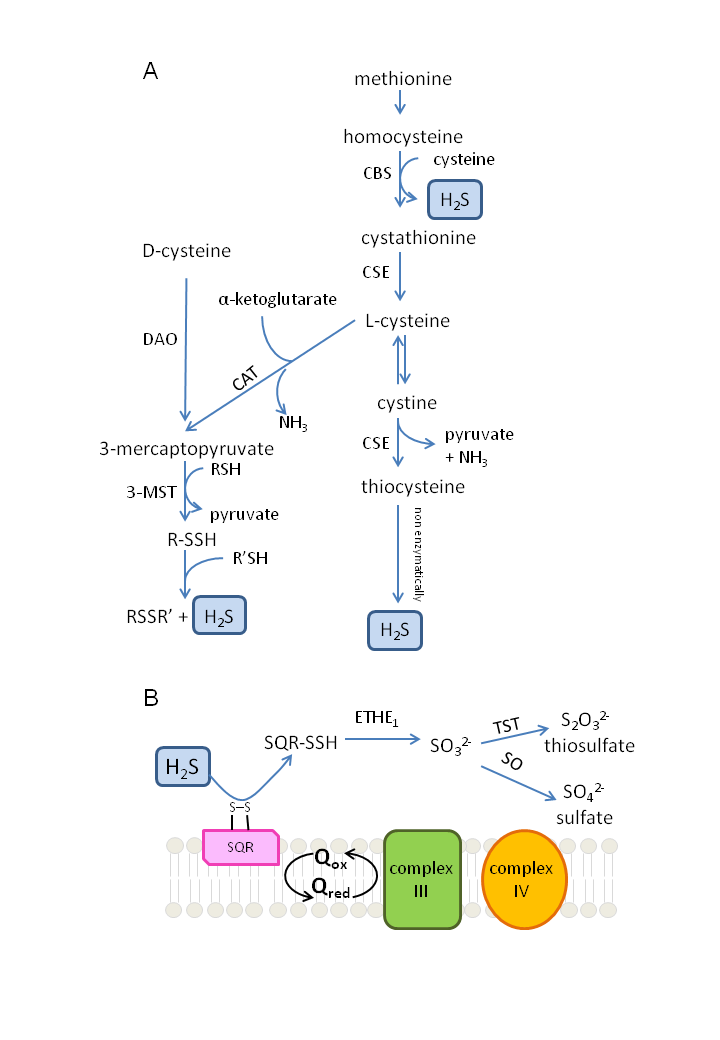

Supplement: S1 Fig — (A) H2S can be produced by three enzymes i.e., cystathionine β-synthase (CBS), cystathionine γ-lyase (CSE) and 3-mercaptopyruvate sulfur transferase (3-MST). CBS and CSE are pryridoxal-5-phosphate-dependent enzymes localized in the cytosol that in the transsulfuration pathway use L-Cys and homocysteine (Hcy) as substrates for H2S production [Kabil and Banerjee 2010; Singh and Banerjee 2011]. Hcy, which is formed from methionine in the methionine cycle, in the reaction catalyzed by CBS produces cystathionine at the same time releasing a H2S molecule. Next, CSE converts cystathionine into L-Cys that is spontaneously oxidized to cystine. As a substrate for CSE, L-Cys is further metabolized to L-cysteine persulfide known as thiocysteine from which H2S is generated in a non-enzymatic manner. The third enzyme 3-MST, synthesizes H2S from 3-mercaptopyruvate (3-MP) which is formed from L-Cys in a reaction catalyzed by cysteine aminotransferase (CAT) or from D-cysteine via D-amino acid oxidase (DAO). 3-MST and CAT are ubiquitous enzymes which are localized both in the mitochondria and cytosol, while the occurrence of DAO is only restricted to the kidney and brain peroxisomes [76]. The 3-MST-catalyzed reaction requires a reducing agent (RSH) to release H2S from persulfide (R-SSH) formed from the substrate, 3-MP [Kabil and Banerjee 2010; Nagahara et al. 2007]. (B) In the first step, H2S is oxidized by mitochondrial membrane-bound flavoprotein, sulfide quinone oxidoreductase (SQR), which forms a protein-bound persulfide while electrons are transferred to ubiquinone (Qox). In the next steps, a sulfur dioxigenase (ETHE1) oxidizes SQR-bound sulfane sulfur to sulfite (SO32-), which is subsequently converted to thiosulfate (S2O32-) by the transfer of a second persulfide equivalent catalyzed by the rhodanese (TST) [73]. Alternatively, SO32- can be oxidized to sulfate (SO42-) by sulfite oxidase (SO). (TIF) [file pone.0147238.s001.TIF]
